# Supplementary material for: Lamina-associated polypeptide 2α is required for intranuclear MRTF-A activity
Source: Sci Rep. 2022 Feb 10;12:2306. doi: 10.1038/s41598-022-06135-5 (PMC8831594; doi:10.1038/s41598-022-06135-5)

Supplementary

Figure S1. Related to figure 1.

(a) Western blot showing depletion of Lap2α and Lap2ß in NIH 3T3 with siRNA against total Lap2. Used siRNA on the top, molecular weight markers on the left, and antibody used in Western Blot on the right.

(b) Venn diagram showing overlap of serum-responsive genes in control and Lap2-depleted NIH 3T3 fibroblasts.

(c) Violin plot showing distribution of log2FC of genes activated in control and Lap2α-depleted NIH 3T3 fibroblasts. N=356. Statistically significant differences (*) tested by Mann-Whitney U-test, $P<0.0001$.

(d) SRF and MRTF-A average normalized read counts (FPKM) on the SRF peaks identified in serum-stimulated conditions in NIH 3T3 fibroblasts. Metaprofiles are centered on SRF in 15% peak summits.

(e) Violin plots showing distribution of SRF and MRTF-A average normalized read counts (FPKM) at identified SRF in 15% binding sites (N=383) at 400 bp region centered on SRF in 15% peak summits. Statistically significant differences (*) tested by Mann-Whitney U test $P < 0.00001$.

(f) Heat maps showing enrichment of SRF and MRTF-A at identified SRF binding sites in 15% (N=383) across a 5-kb region centered on the peak summit and sorted according to decreasing MRTF-A in 15% normalized read count. The read count are normalized to input signal accordingly.

(g) Western blot showing depletion of Lap2α in NIH 3T3; data shown as in A.

(h) Western blot showing depletion of Lap2β in NIH 3T3; data shown as in A

(i) Western blot showing Lap2α and Lap2β protein amounts in Lap2α WT and KO mouse dermal fibroblasts

Figure S3. Lap2α is not required for MRTF-A nuclear localization; related to figure 3.

(a) Localization of endogenous MRTF-A in WT and Lap2α KO MDF cells after 45 min of serum (15%) stimulation. Scale bars 10 µm.

(b) Localization of MRTF-A-GFP in serum-starved (0.3% FBS), and serum-stimulated (15% FBS) NIH 3T3 fibroblasts transfected with control (upper panel) or Lap2α (lower panel) siRNAs. Scale bars 10 µm.

(c) Localization of endogenous MRTF-A in serum-starved (0.3% FBS), serum-stimulated (30 min, 15% FBS), Leptomycin B (LMB)-treated (30 min, 20 nM) and cytochalasin D (CD)-treated (30 min, 2µM) Lap2α WT (upper panel) and KO (lower panel) MDF cells. Scale bars 10 µm.

(d) Quantification of endogenous MRTF-A localization in Lap2α WT (left) and KO (right) MDFs. Number of cells: N=154 WT 0.3%; N=141 WT 15%; N=158 WT LMB; N=129 WT CD; N=195 KO 0.3%; N=178 KO 15%; N=231 KO LMB; N=52 KO CD

Figure S4. Chromatin binding of MRTF-A and Pol II in Lap2α WT and KO cells, related to figure 4.

(a) Venn diagrams showing overlaps of MRTF-A peaks enriched with SRF motif in Lap2α WT and KO MDF in serum-starved (0.3% FBS) and serum-stimulated (15% FBS) conditions.

(b) Heat map showing enrichment of MRTF-A at identified binding sites (N=155) in 15% serum across a 5 kb region centered on the peak summit and sorted according to decreasing MRTF-A in 15% serum in KO normalized read count. The read counts are normalized to input signal accordingly.

(c) MRTF-A coverage on selected 59 peaks with MRTF-A peaks in Lap2α WT and KO MDF. Metaprofiles with average normalized fragment counts across gene loci, standardized to the same gene length.

(d) Normalized read counts (FPKM) at identified MRTF-A binding sites (N=63) at 400 bp in Lap2α WT vs KO cells in serum-starved (0.3%) and serum-stimulated (15%) conditions. Statistically significant differences (*) tested by Mann-Whitney U test $P < 0.00001$.

(e) Pol II S5P coverage on selected 58 genes in Lap2α WT and KO MDF. Metaprofiles with average normalized fragment counts across gene loci, standardized to the same gene length.

(f) Normalized read counts (FPKM) ratio (in serum-stimulated (15%) vs serum-starved (0.3%) conditions) of Pol II S5P at gene bodies (N=58) in Lap2α WT vs KO cells. Statistically significant differences (*) tested by Mann-Whitney U test $P < 0.00001$.

Figure S5. Distribution of active histone marks (H3K4me3 and H3K9Ac) on up- and down-regulated genes in Lap2α KO vs WT MDF, related to figure 5.

Reanalyzed from Gesson et al, 2016.

(a, b) Histone marks H3K4me3 (a) and H3K9Ac (b) at the TSS of all genes in WT and Lap2α KO cells. Metaprofiles show 5 kb window with average normalized fragment counts centered on TSS.

(c, d) Histone marks H3K9Ac (c) and H3K4me3 (d) at the TSS of genes up-regulated in Lap2α KO fibroblasts is higher in KO cells. Metaprofiles show 5 kb window with average normalized fragment counts centered on TSS**.**

(e, f) Histone marks H3K9Ac (c) and H3K4me3 (d) at the TSS of genes down-regulated in Lap2α KO fibroblasts is higher in WT cells. Metaprofiles show 5 kb window with average normalized fragment counts centered on TSS

Figure S6. Lap2α does not interact with SRF, related to figure 6.

(a) Lap2α does not co-immunoprecipitate SRF. Immunoprecipitation (IP) was performed using anti-Lap2α and anti-IgG (control) antibodies. Immunoprecipitates and cell lysates were subjected to WB analysis using anti-Lap2α, anti-Lamin A (positive control), anti-SRF and anti-BAF (positive control) antibodies. Protein weight markers on the left and the antibody used in Western Blot on the right.

(b) Schematic presentation of MRTF-A and Lap2α constructs used in co-immunoprecipitation and pull-down experiments.

.

Supplementary Figure 1

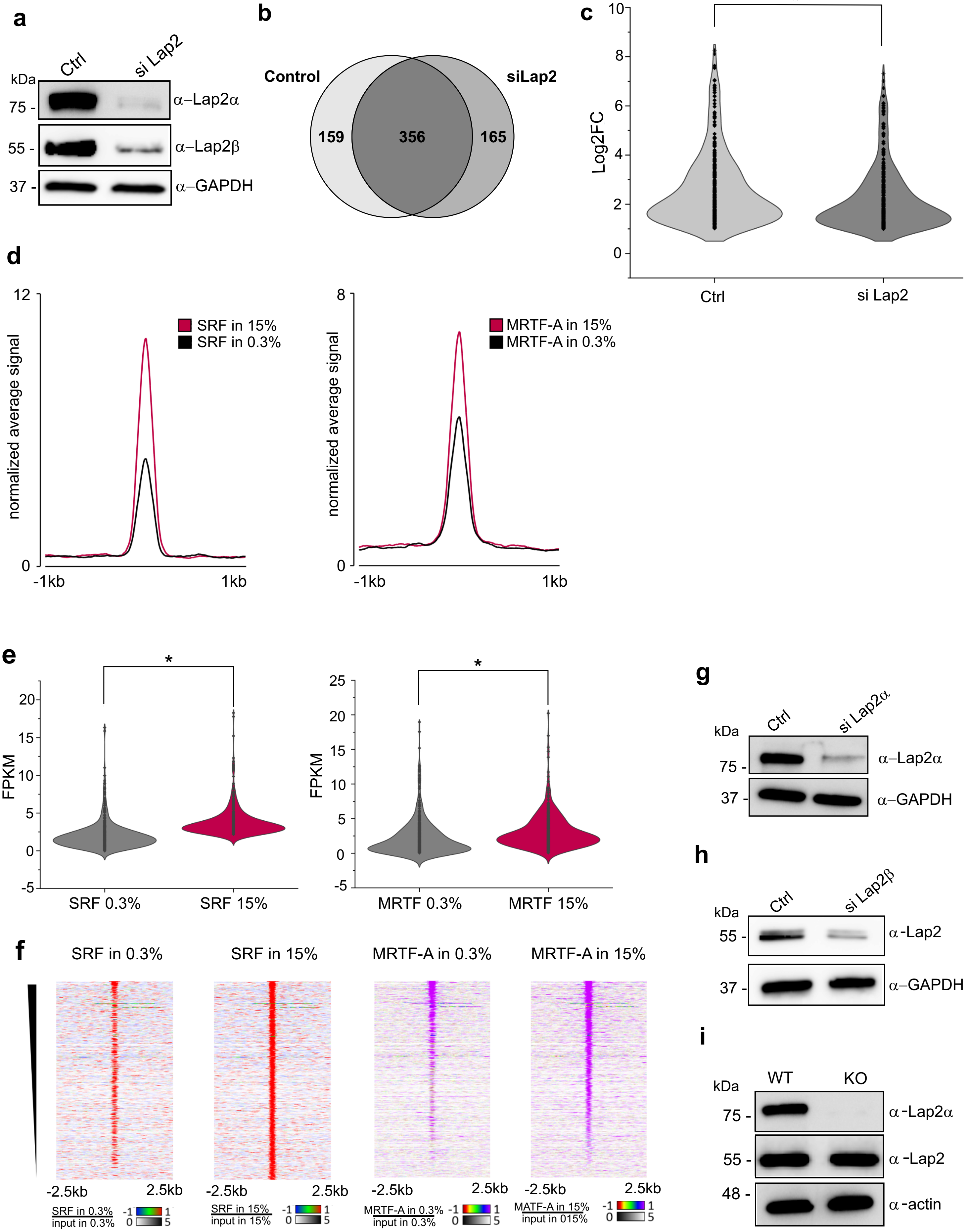

Supplementary Figure 3

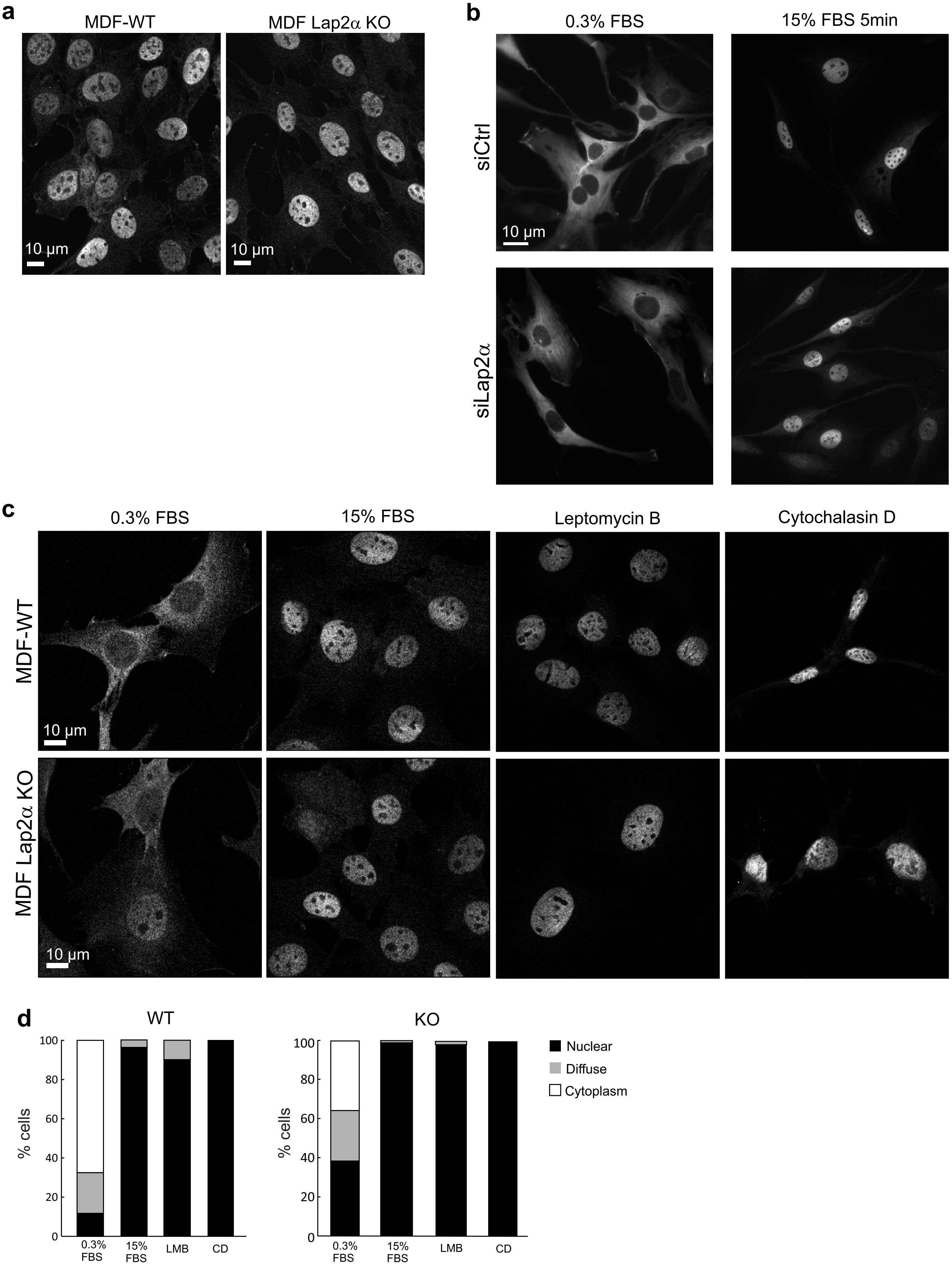

Supplementary Figure 4

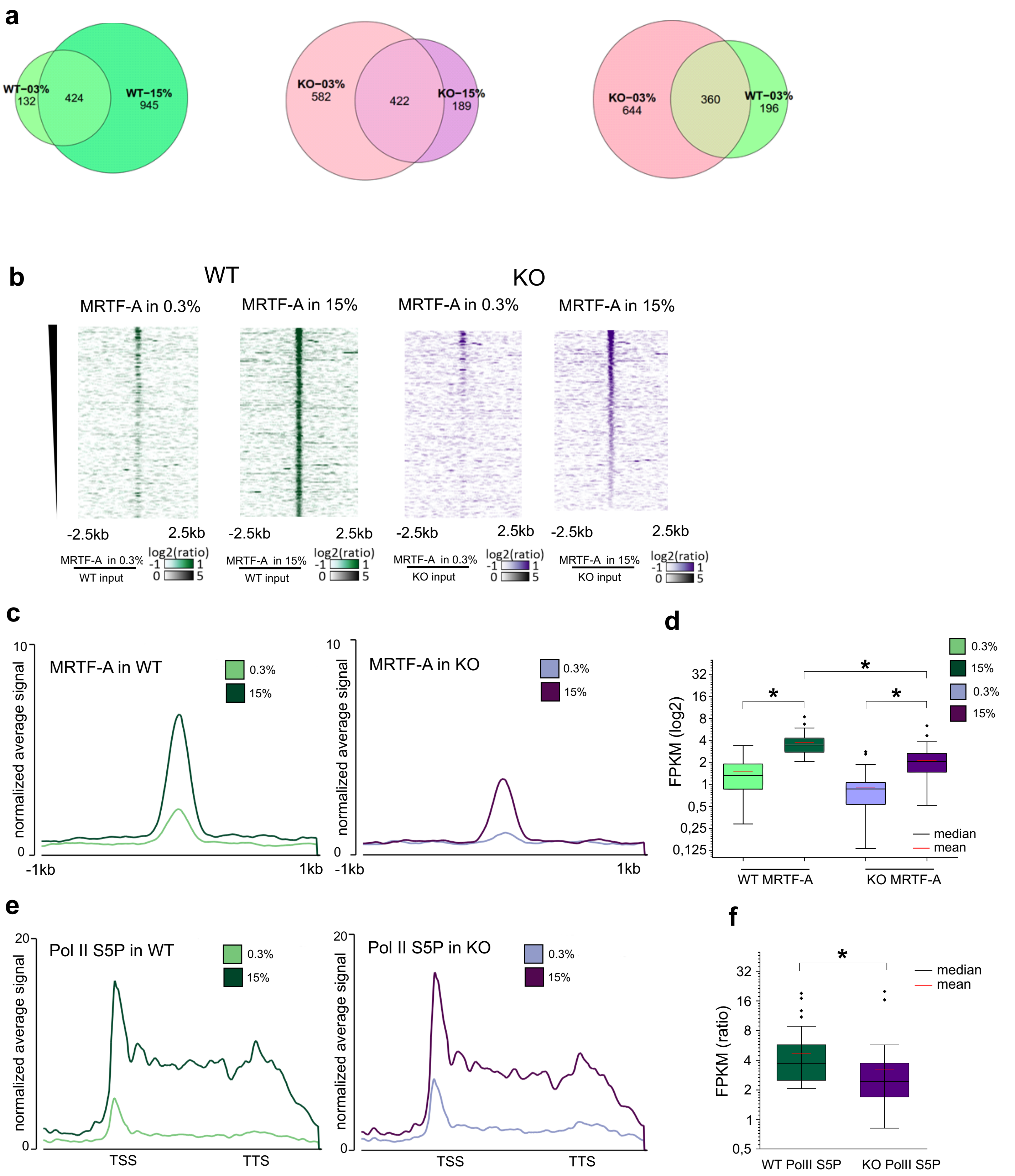

Supplementary Figure 5

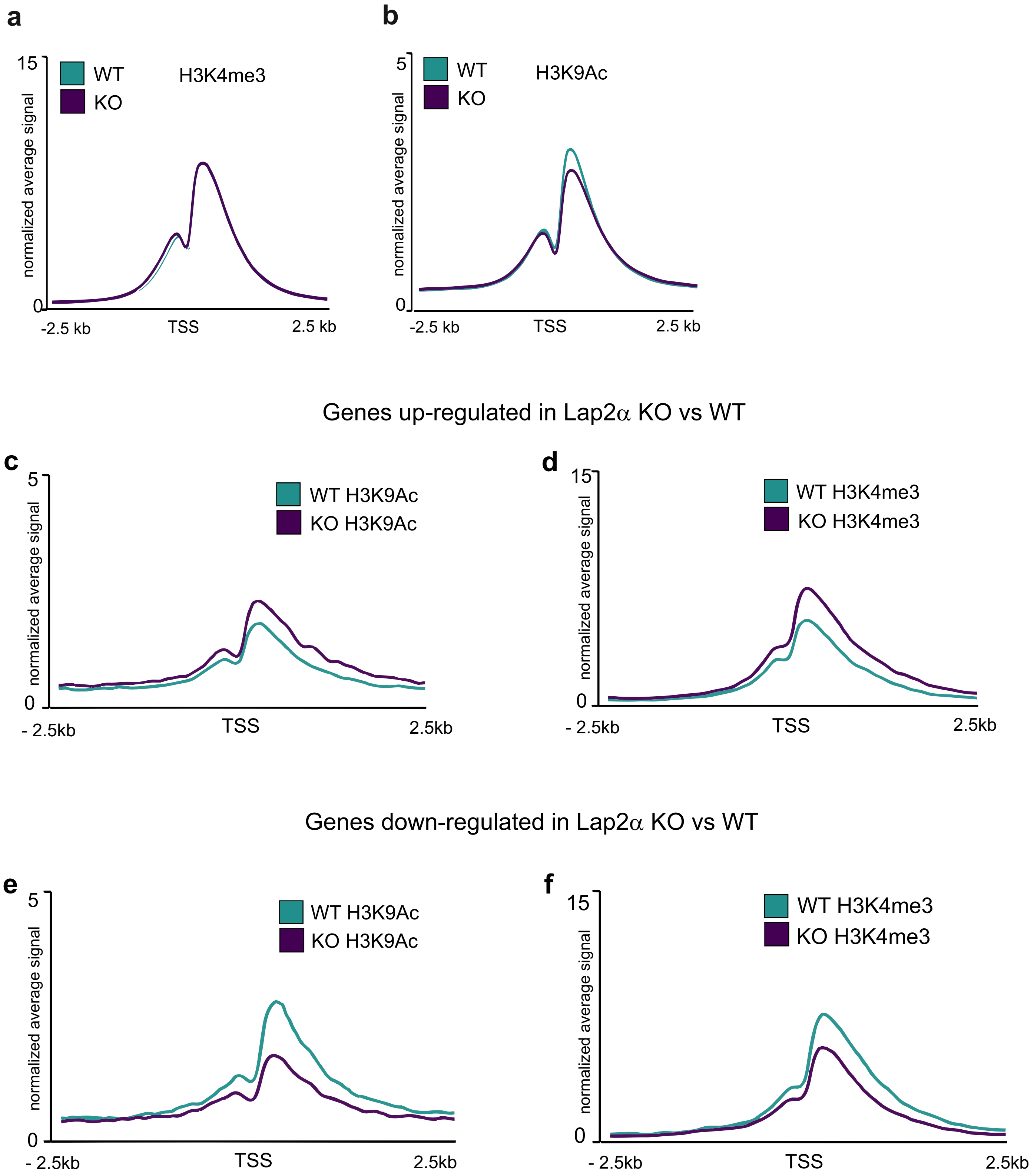

Supplementary Figure 6

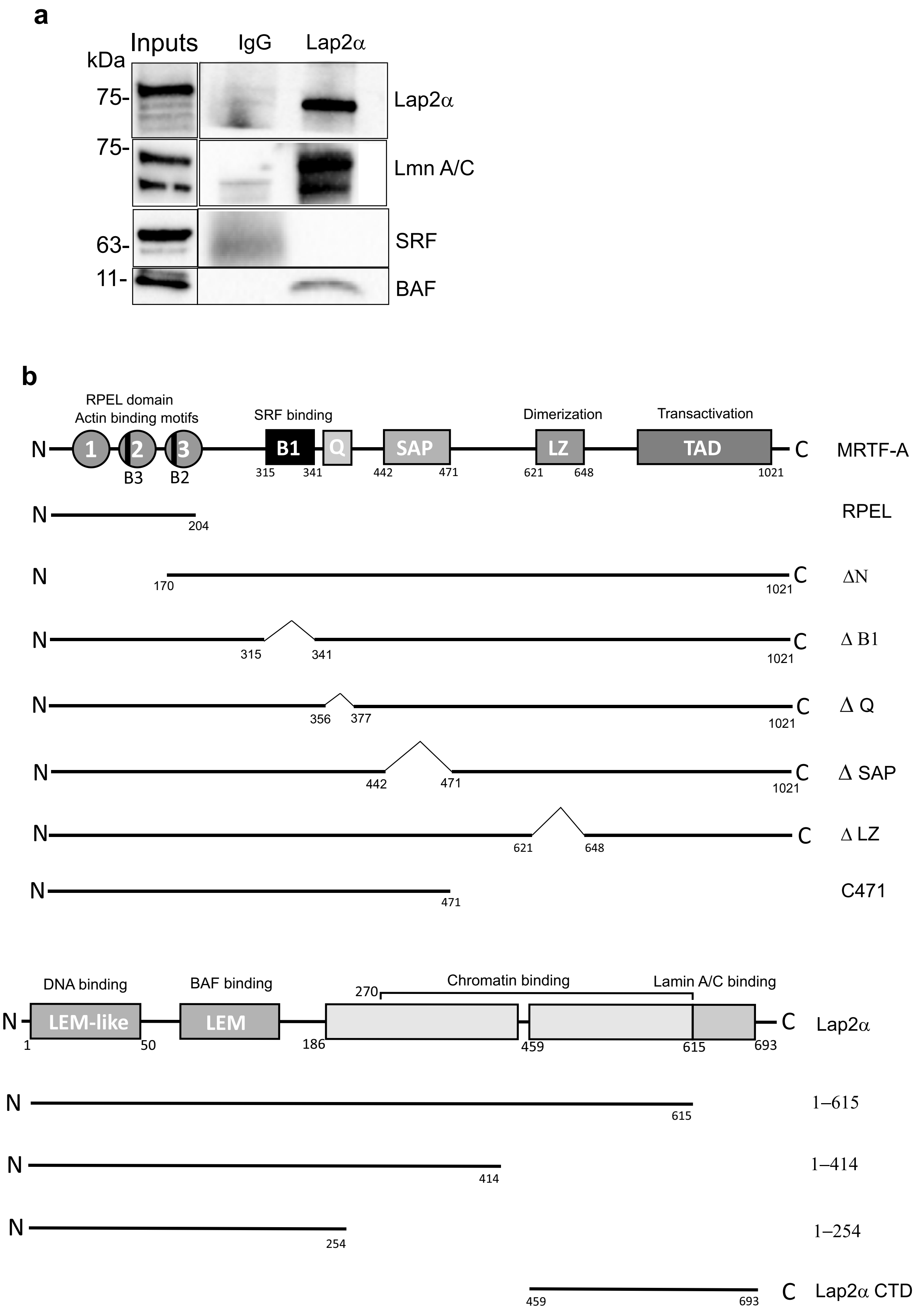

Supplement: Supplementary file 1 — Supplementary Figures. [file 41598_2022_6135_MOESM1_ESM.pdf]
