# Supplementary material for: Lamina-associated polypeptide 2α is required for intranuclear MRTF-A activity
Source: Sci Rep. 2022 Feb 10;12:2306. doi: 10.1038/s41598-022-06135-5 (PMC8831594; doi:10.1038/s41598-022-06135-5)

Figure 6a

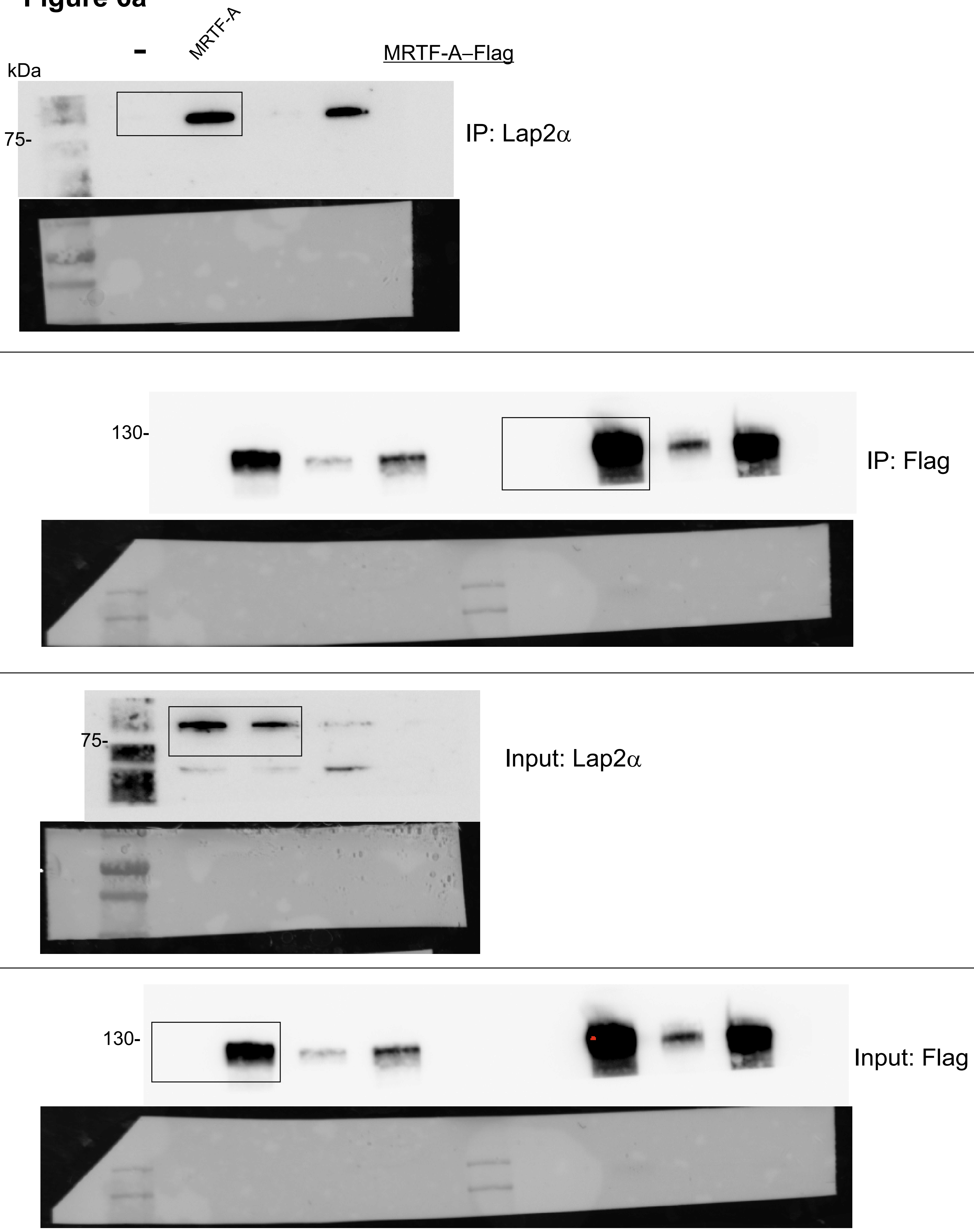

-
MRTF-A
MRTF-A–Flag
kDa
75-
IP: Lap2α
130-
IP: Flag
75-
Input: Lap2α
130-
Input: Flag

Figure 6b

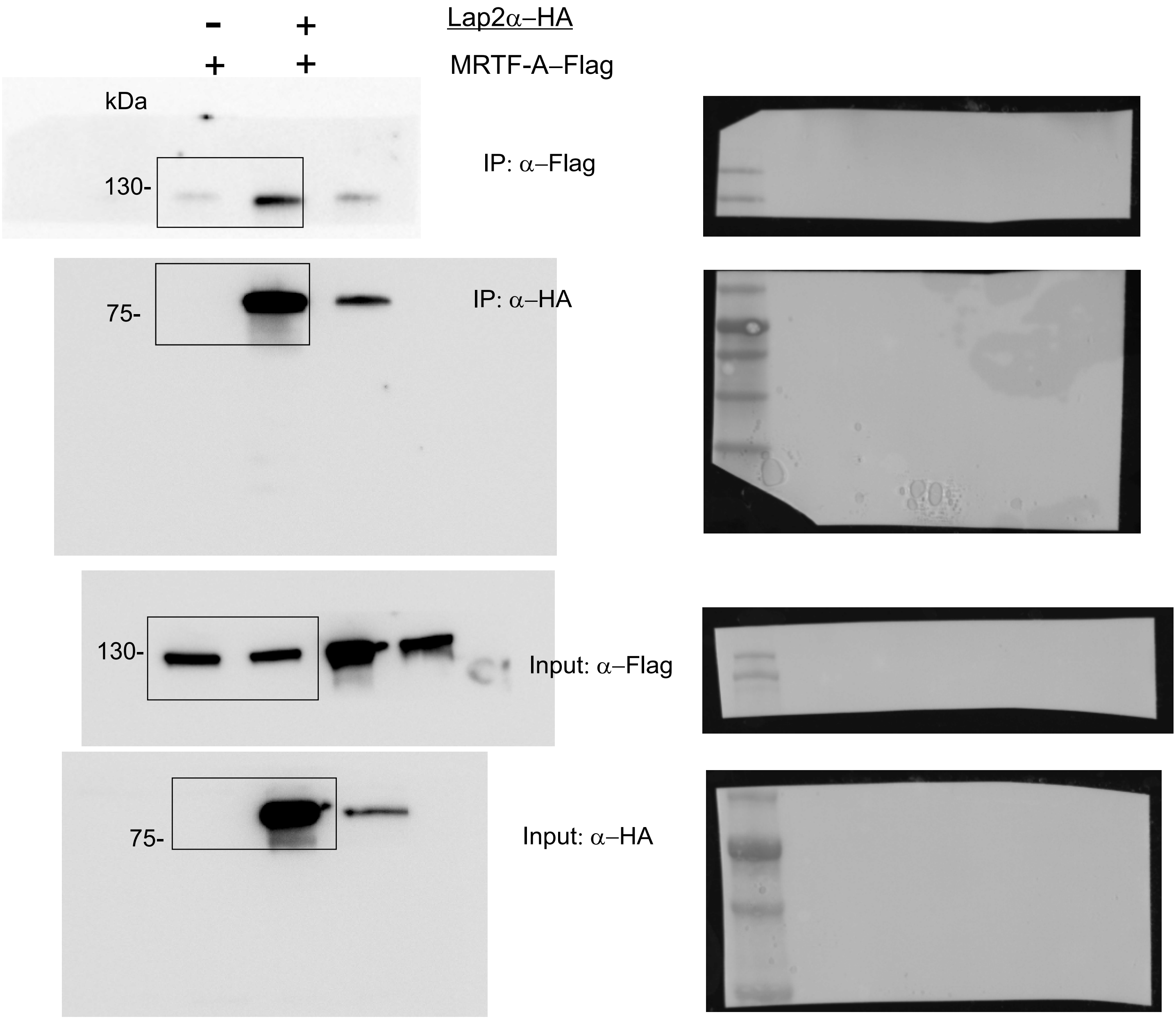

-
+
Lap2α–HA
+
+
MRTF-A–Flag
kDa
130-
IP: α–Flag
75-
IP: α–HA
130-
Input: α–Flag
75-
Input: α–HA

Figure 6c

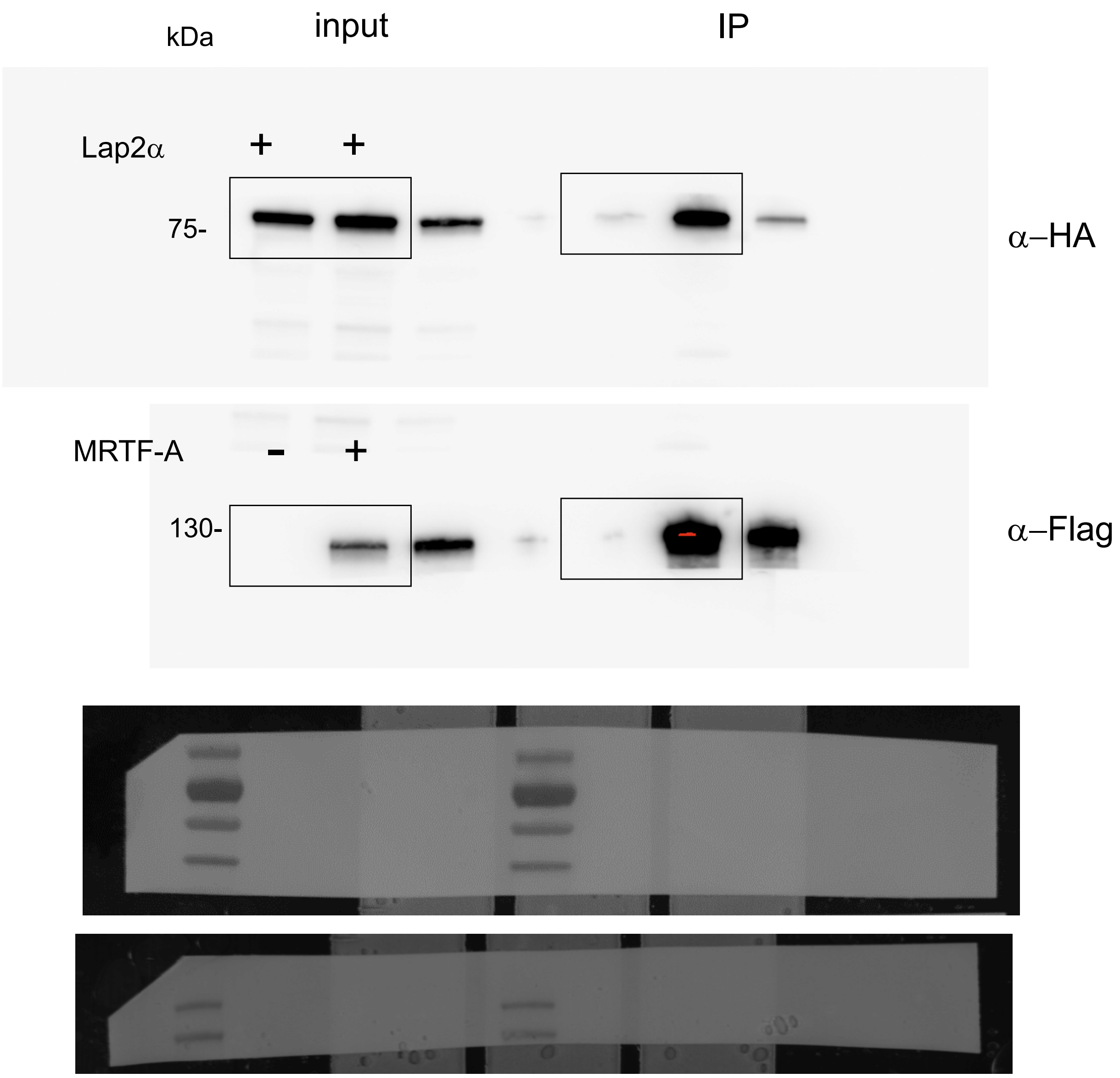

Figure 6e

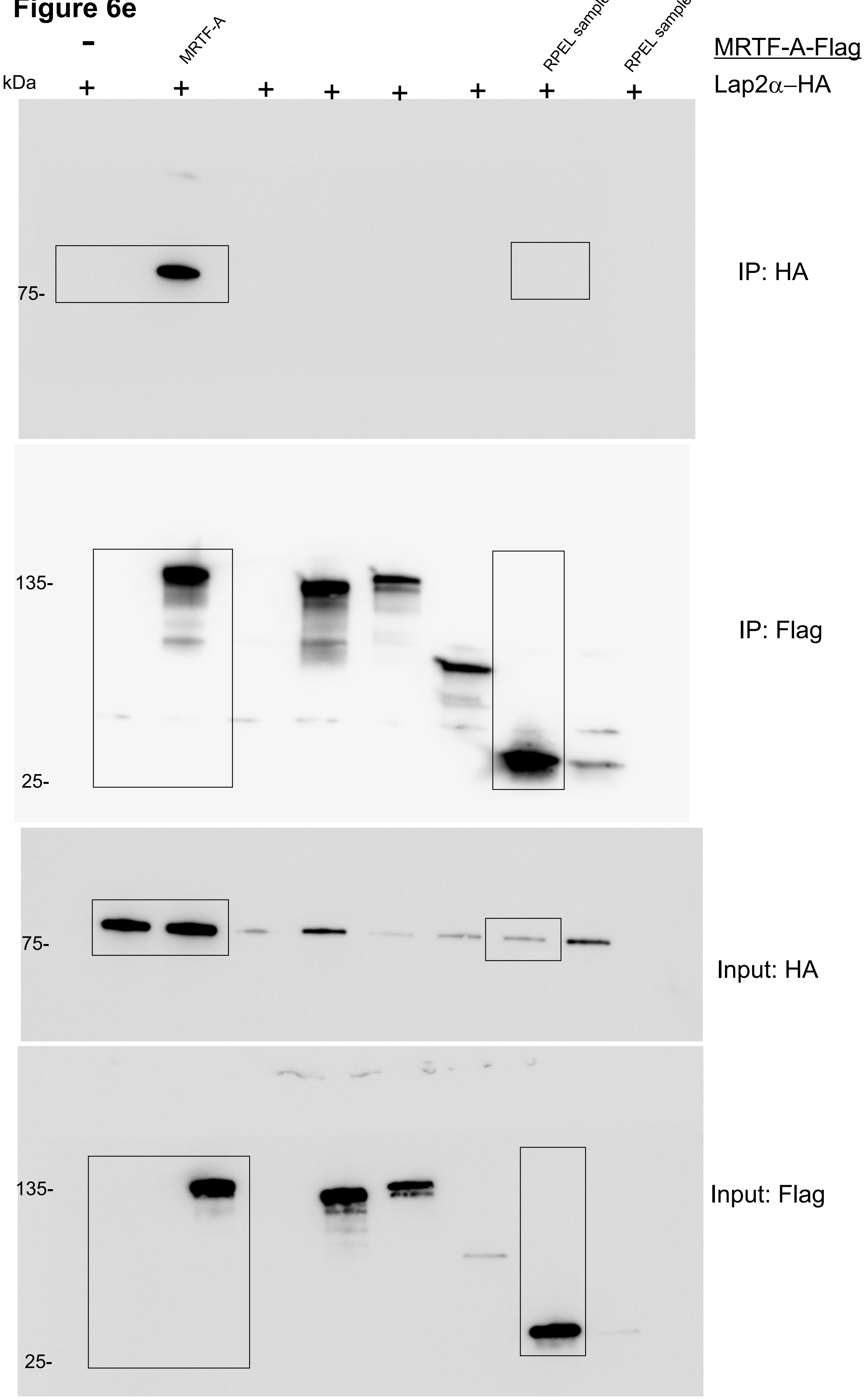
-
MRTF-A
RPEL sample1
RPEL sample2
MRTF-A-Flag
kDa
+ + + + + + + +
Lap2α–HA
75-
IP: HA
135-
25-
IP: Flag
75-
Input: HA
135-
25-
Input: Flag

Figure 6e (continue, size of original membranes)

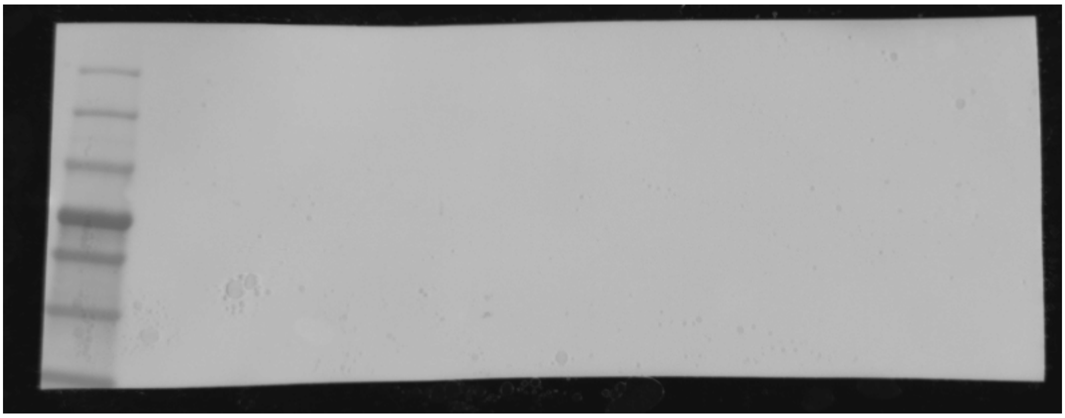

IP: HA

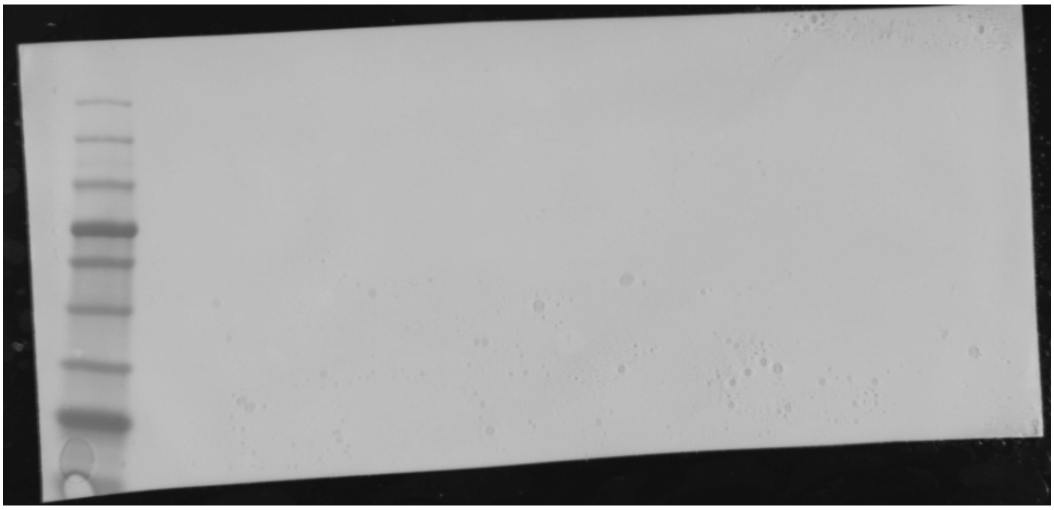

IP: Flag

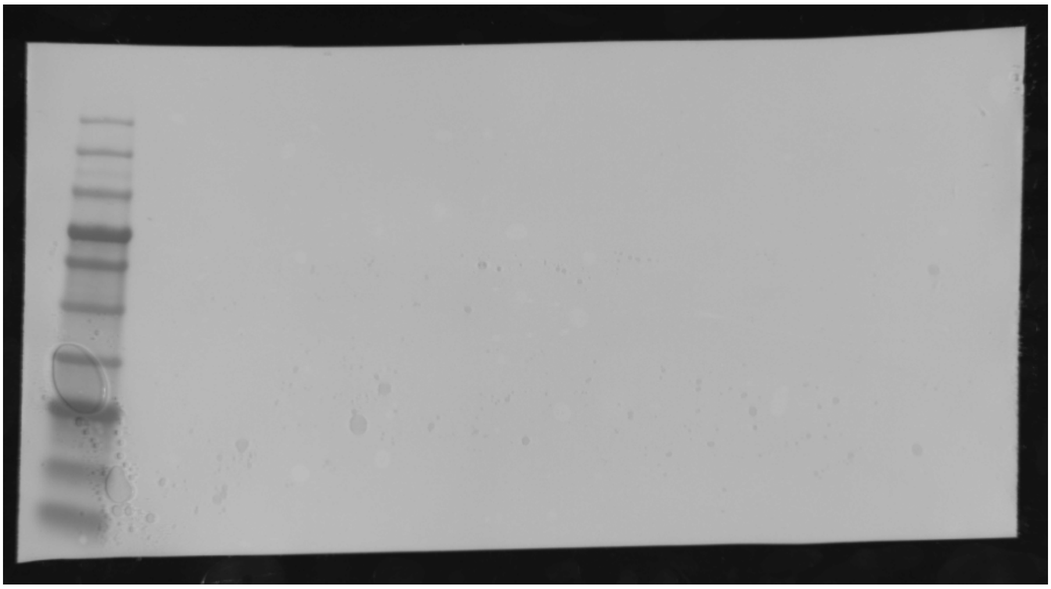

Input: HA

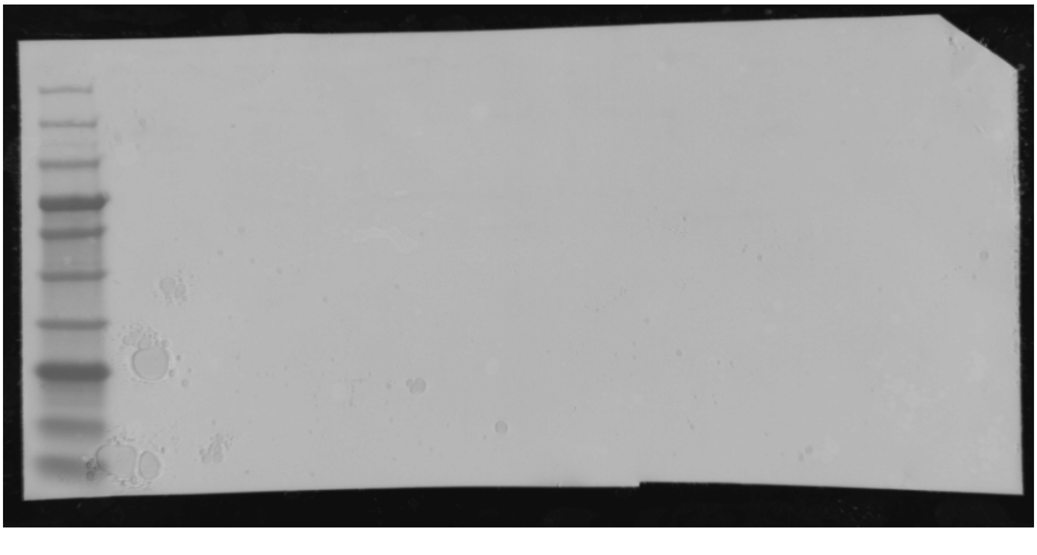

Input: Flag

Figure 6f

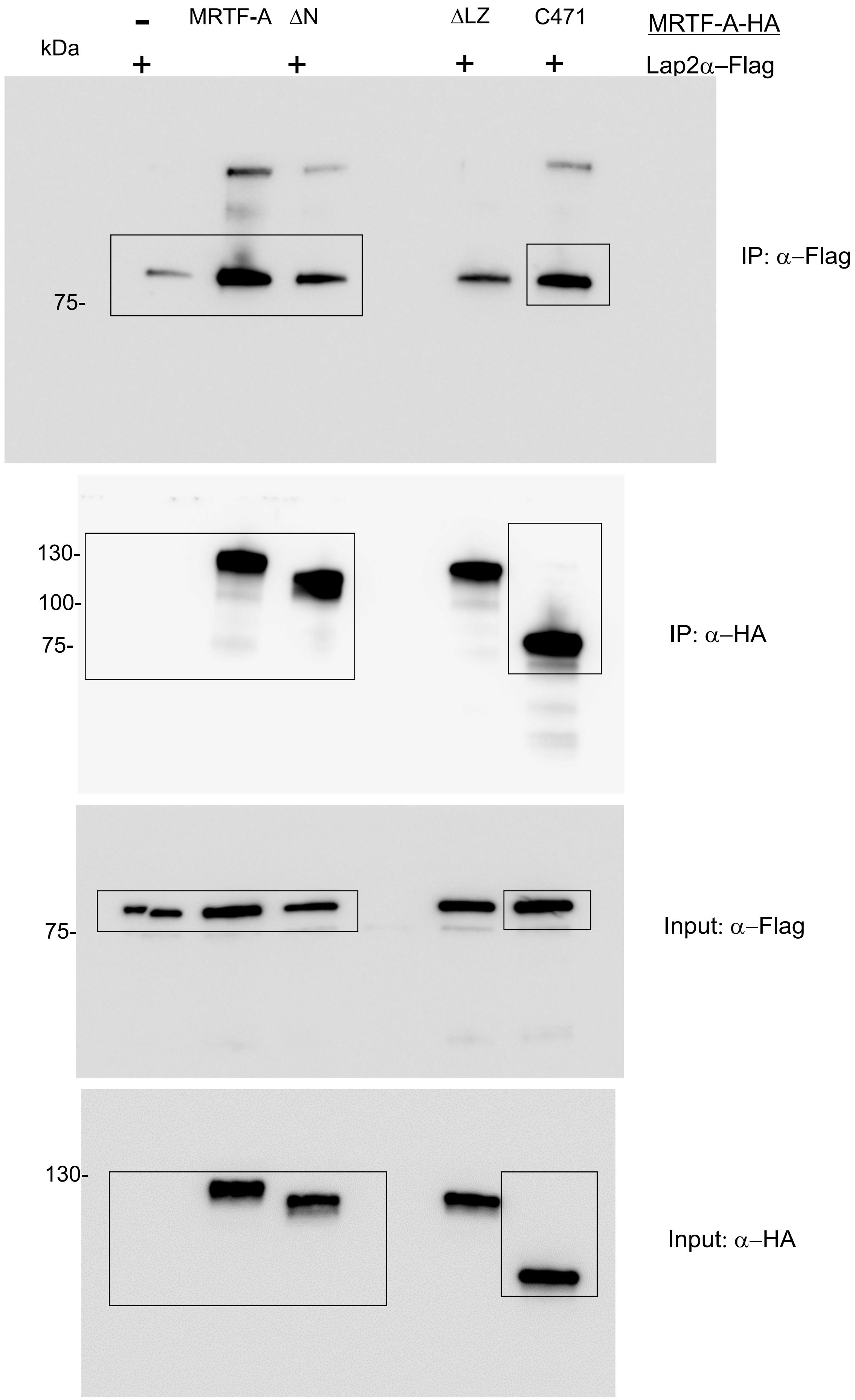

-
MRTF-A
ΔN
ΔLZ
C471
MRTF-A-HA
kDa
+
+
+
+
Lap2α–Flag
75-
IP: α–Flag
130-
100-
75-
IP: α–HA
75-
Input: α–Flag
130-
Input: α–HA

Figure 6f (continue, size of original membranes)

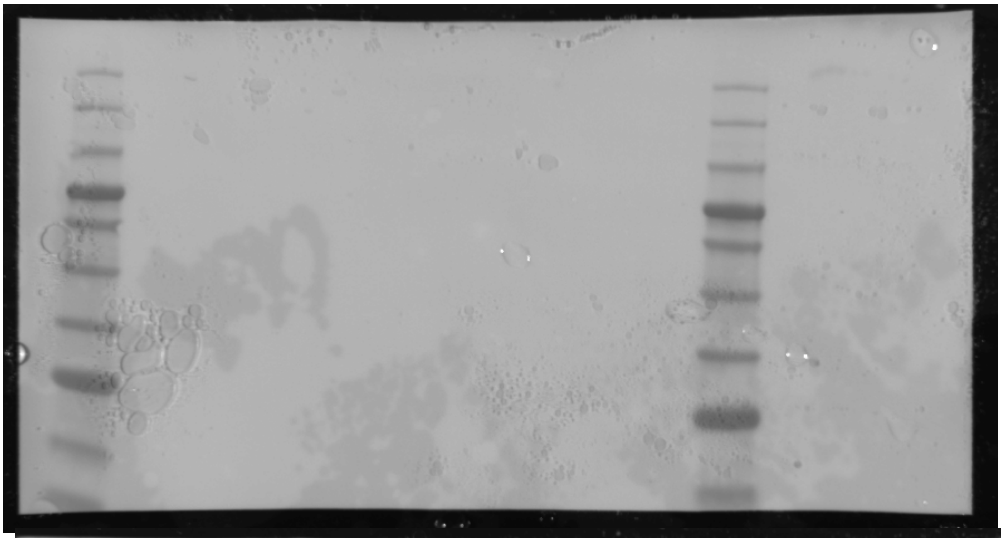

IP: α–Flag

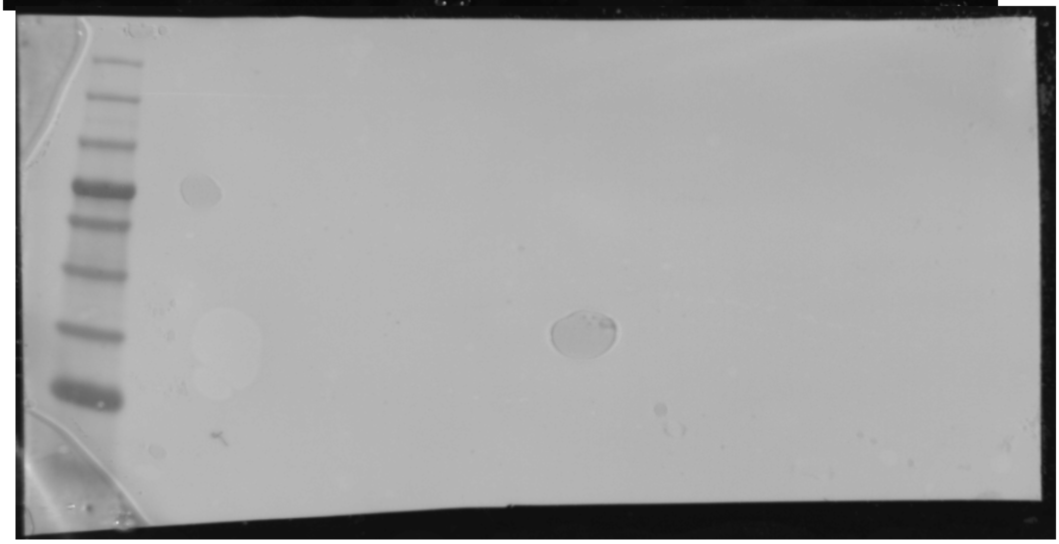

IP: α–HA

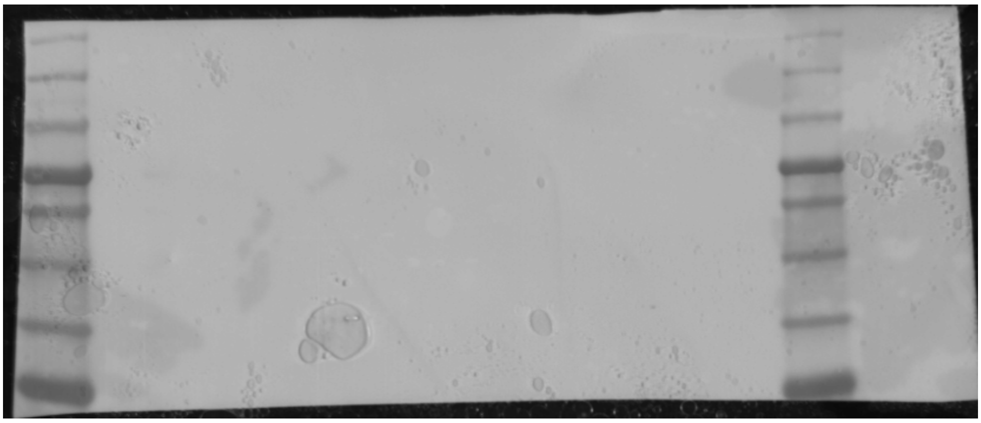

Input: α–Flag

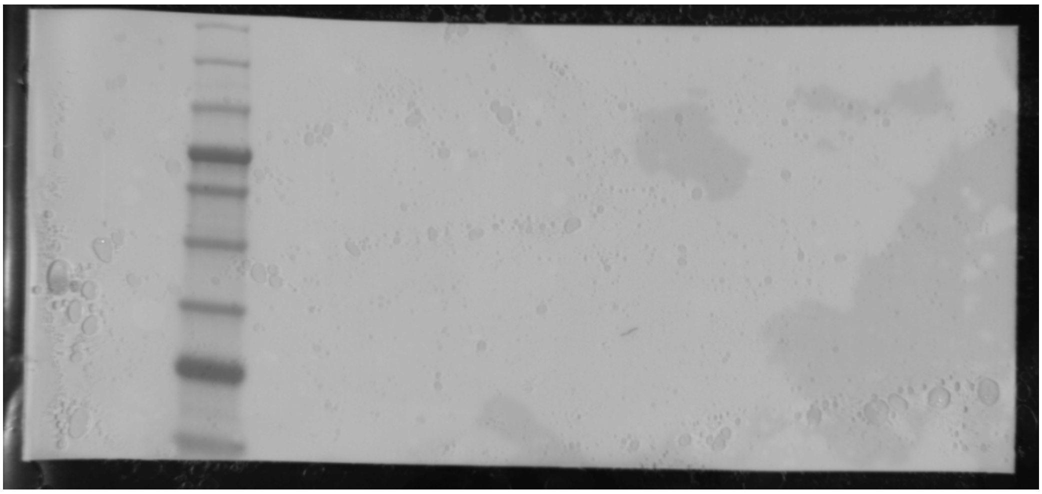

Input: α–HA

Figure 6g

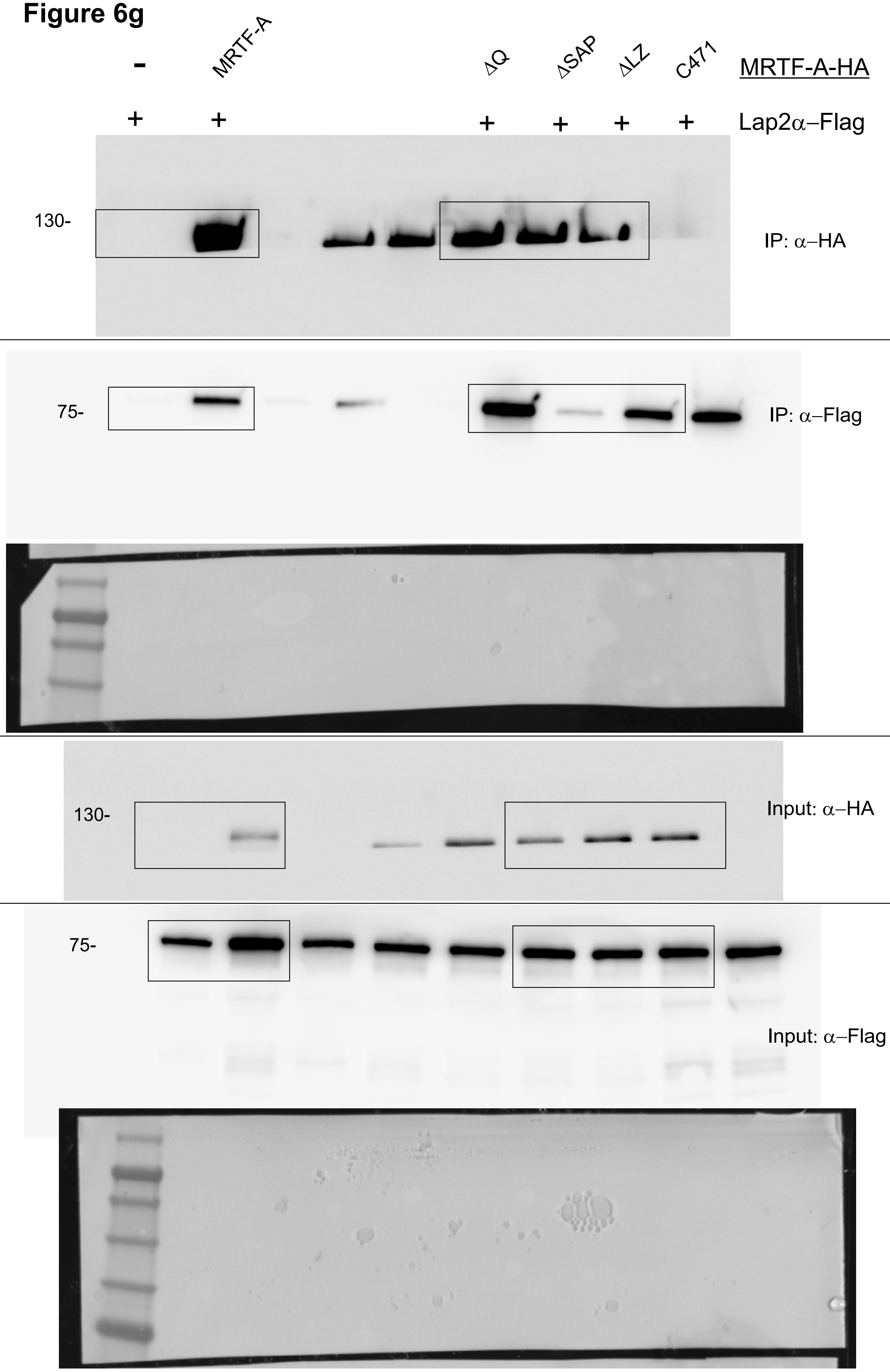

-
MRTF-A
ΔQ
ΔSAP
ΔLZ
C471
MRTF-A-HA
+
+
+
+
+
+
Lap2α–Flag
130-
IP: α–HA
75-
IP: α–Flag
130-
Input: α–HA
75-
Input: α–Flag

Figure 6h

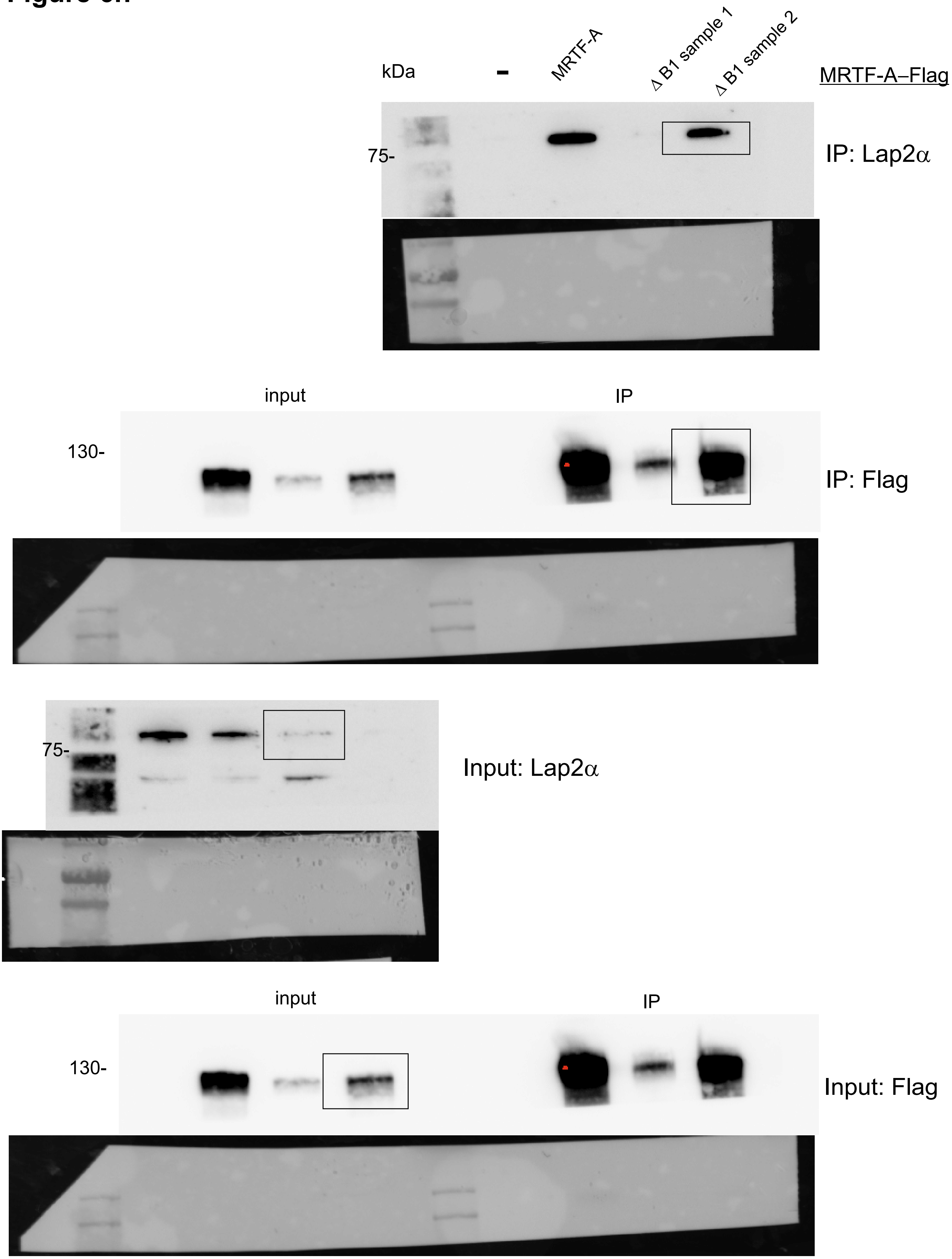

Different amount of Δ B MRTF mutant was used in this CoIP experiment

Figure 6i

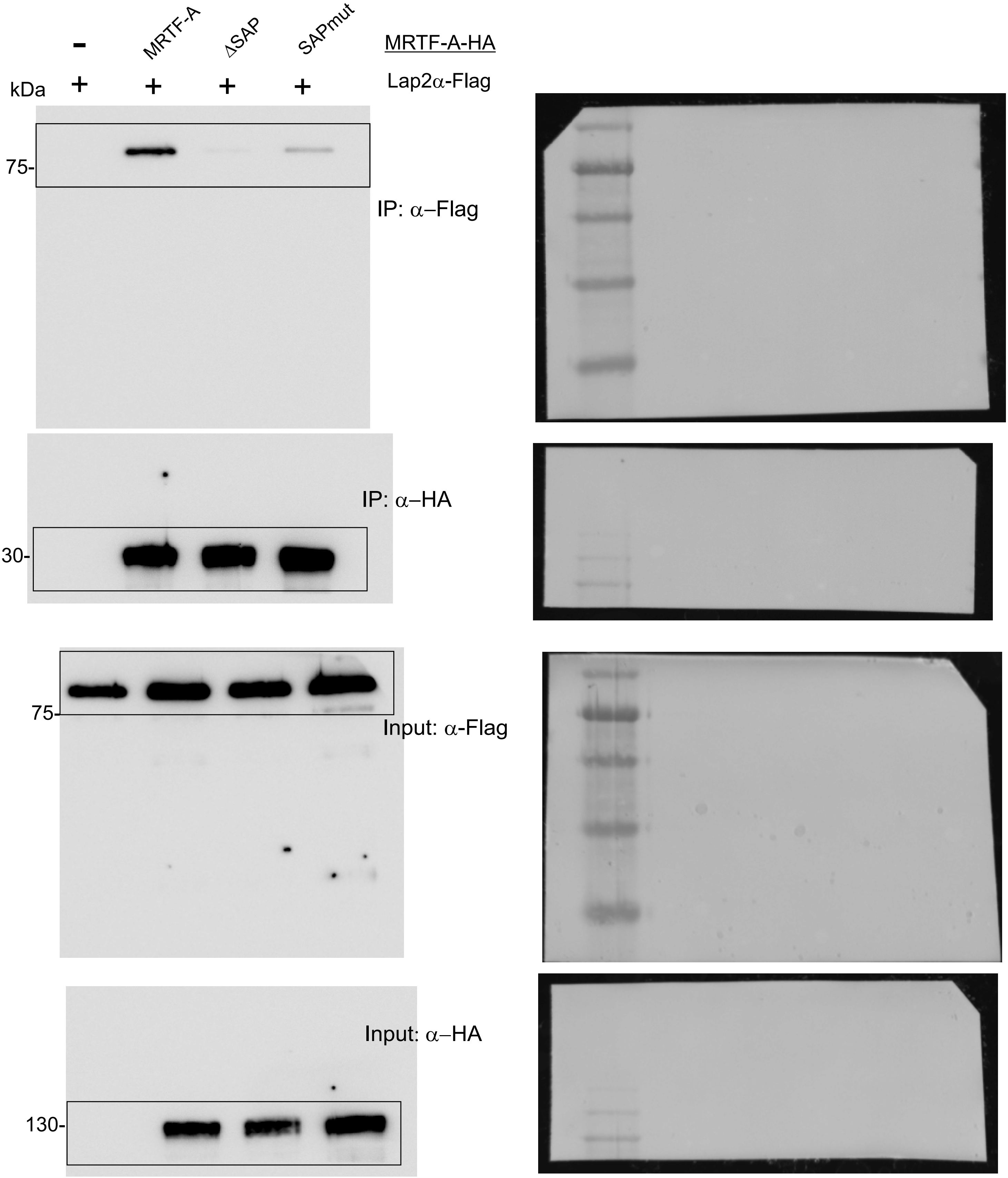
-
MRTF-A
ΔSAP
SAPmut
MRTF-A-HA
kDa
+
+
+
+
Lap2α-Flag
75-
IP: α–Flag
IP: α–HA
130-
75-
Input: α-Flag
Input: α–HA
130-

Figure 6j

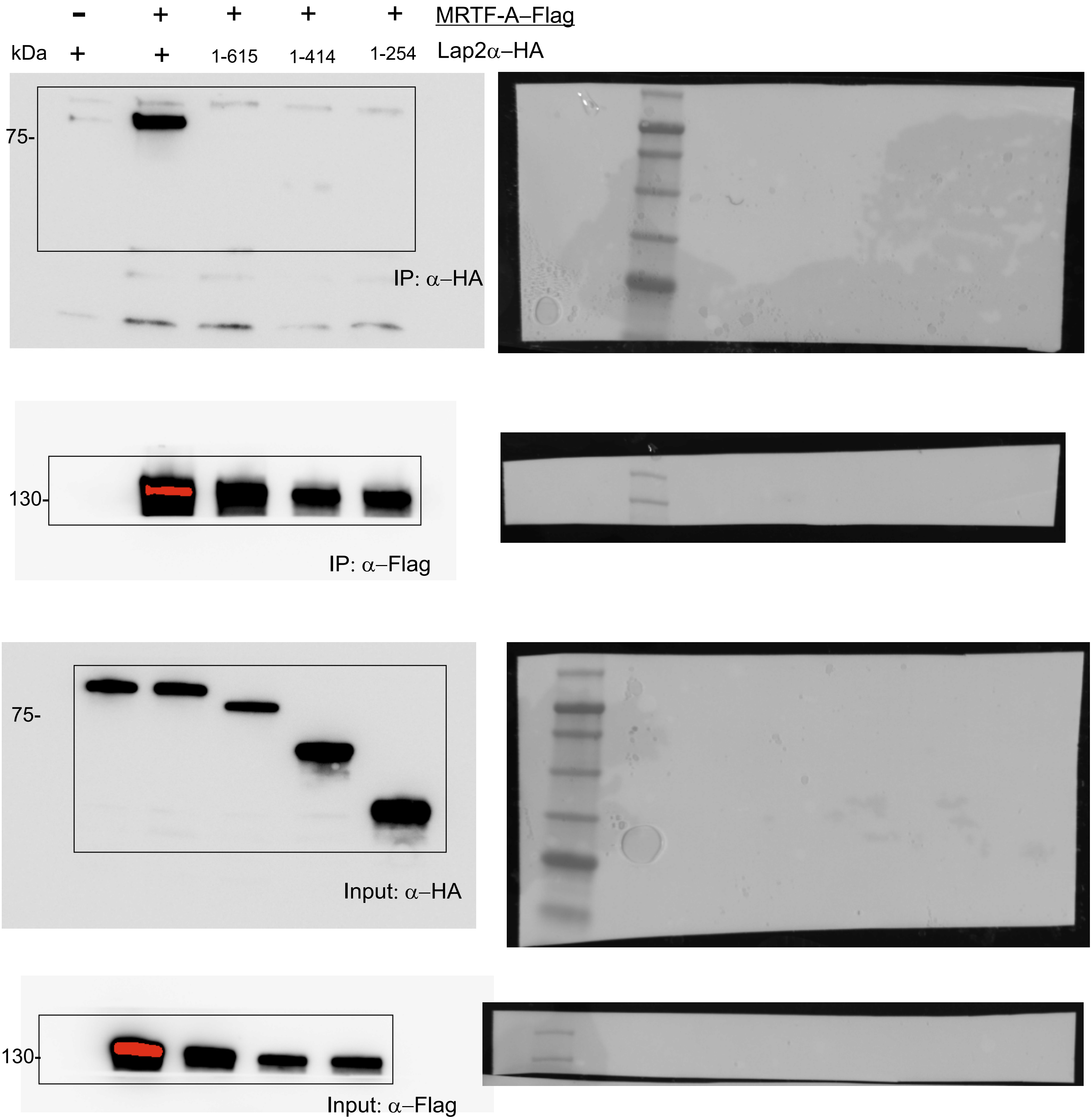

MRTF-A–Flag
Lap2α–HA
- + + + +
+ + 1-615 1-414 1-254
kDa
75-
IP: α–HA
130-
IP: α–Flag
75-
Input: α–HA
130-
Input: α–Flag

Figure 6k

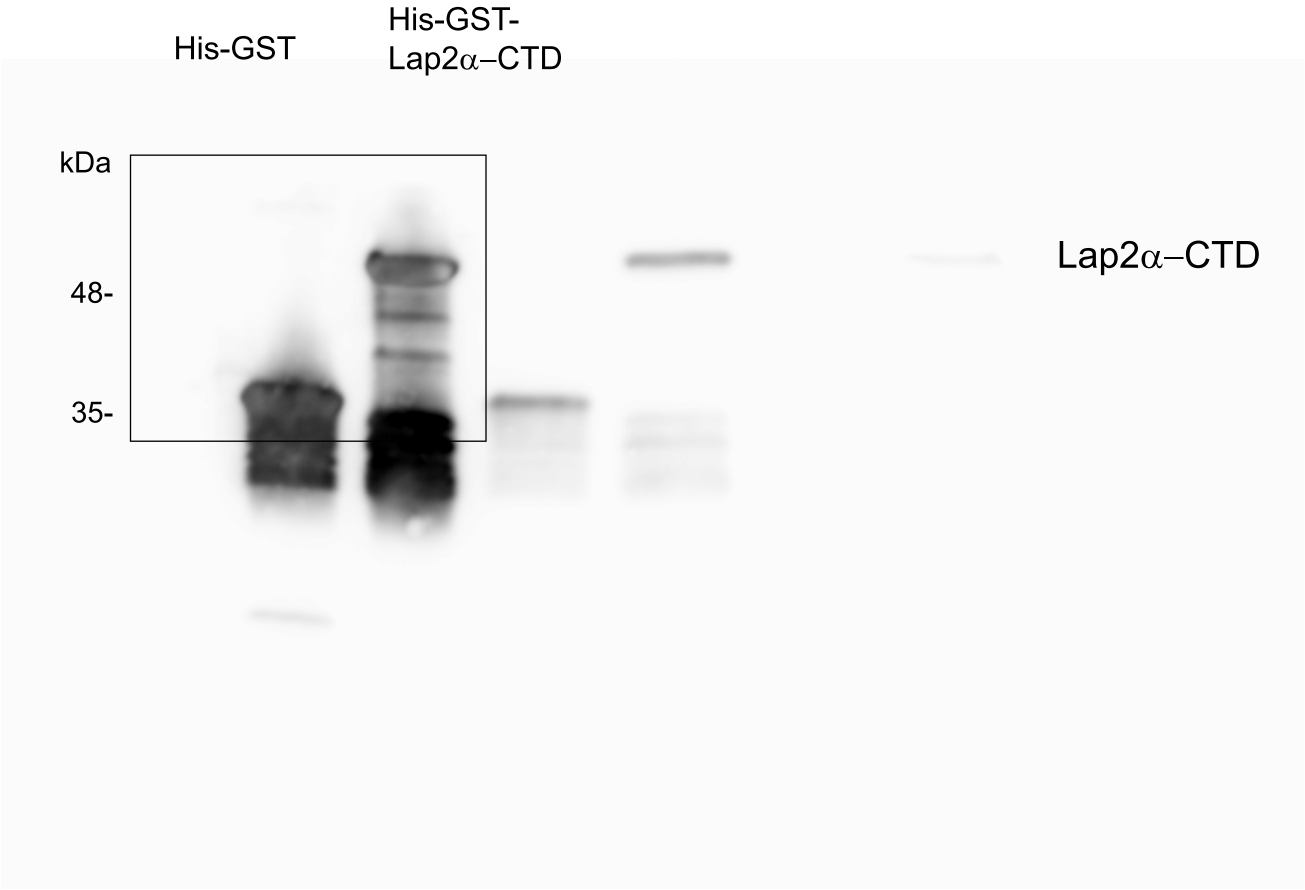

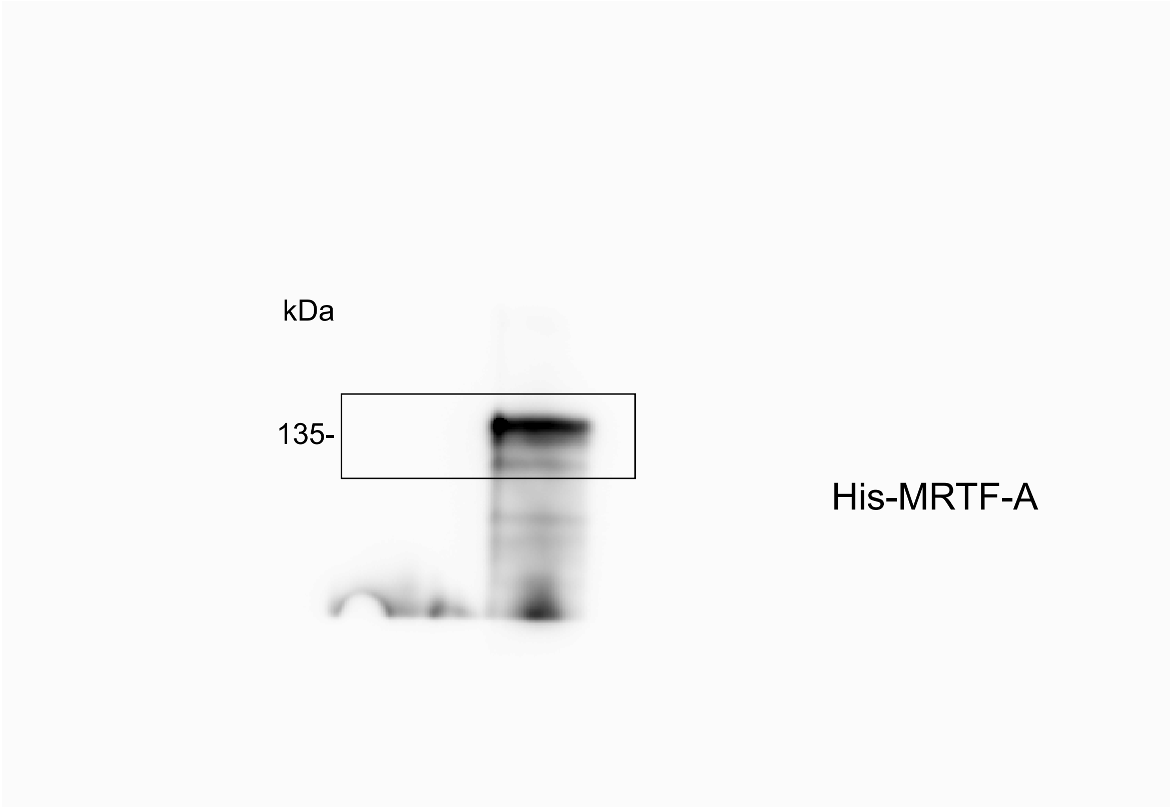

Supplementary 1a

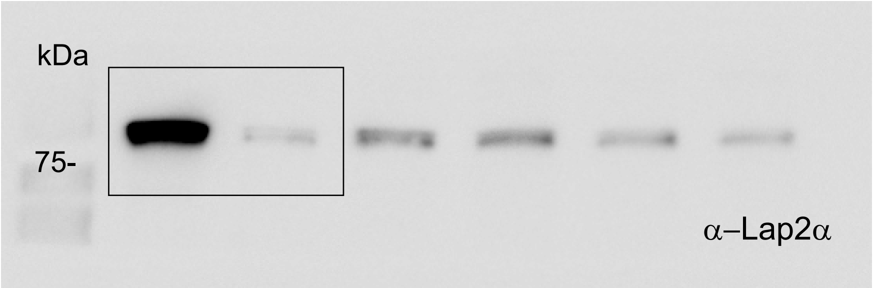

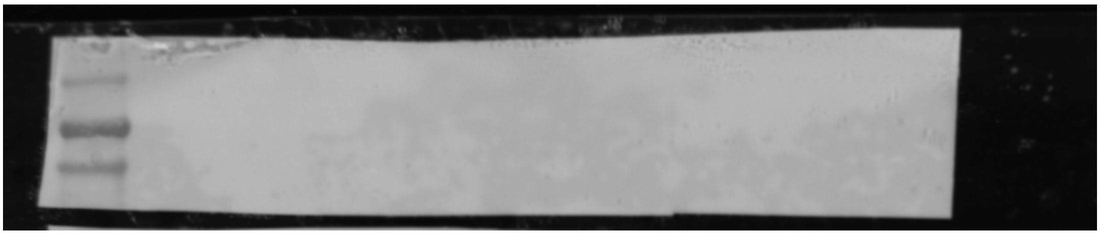

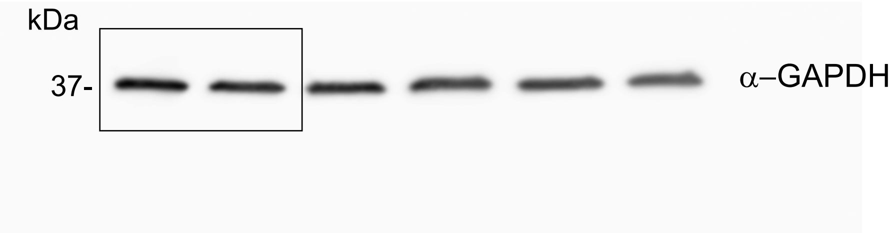

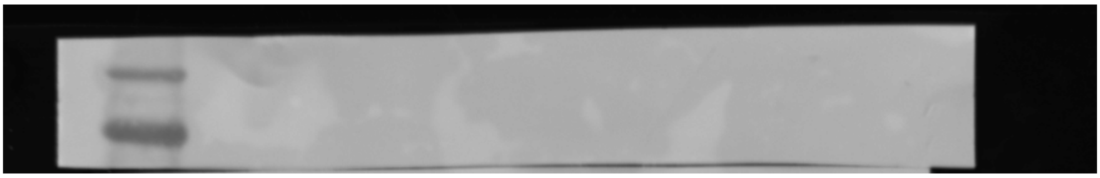

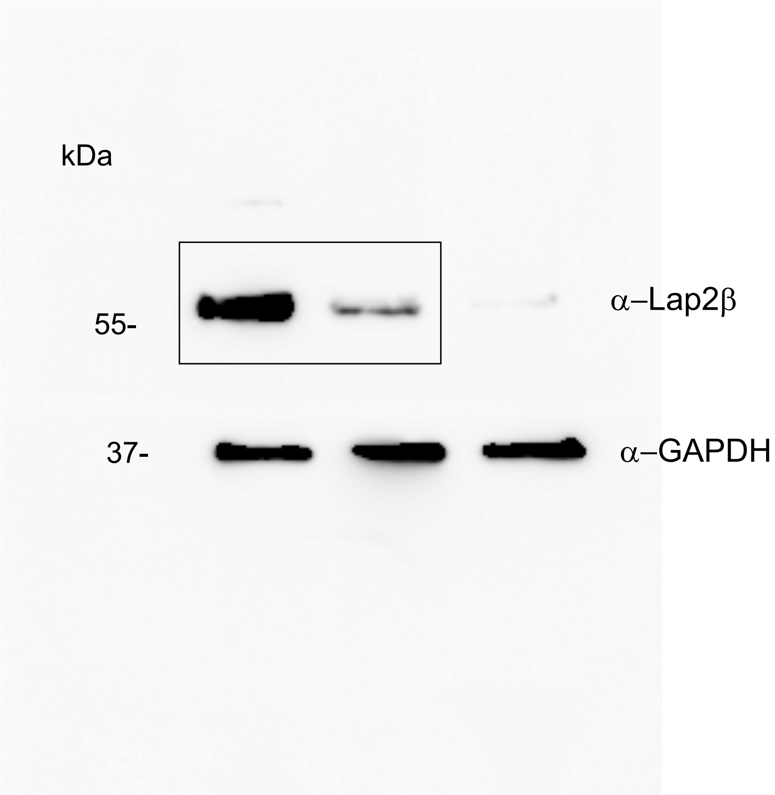

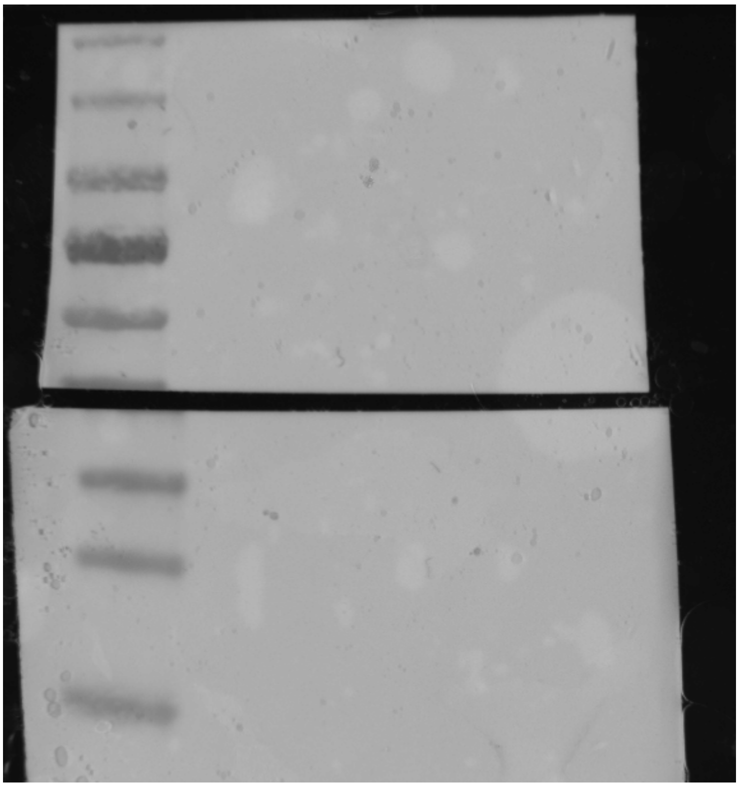

Supplementary 1g

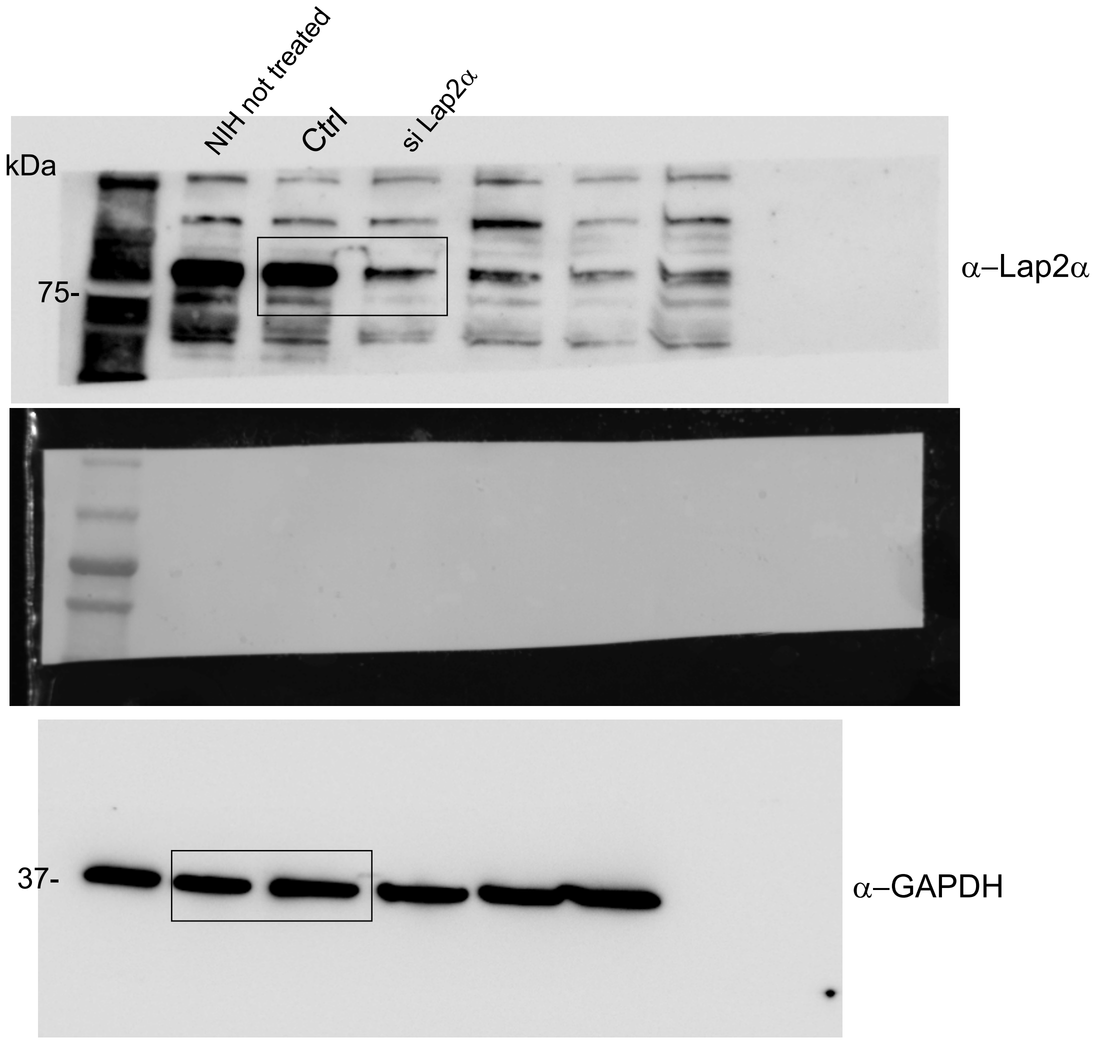

Supplementary 1h

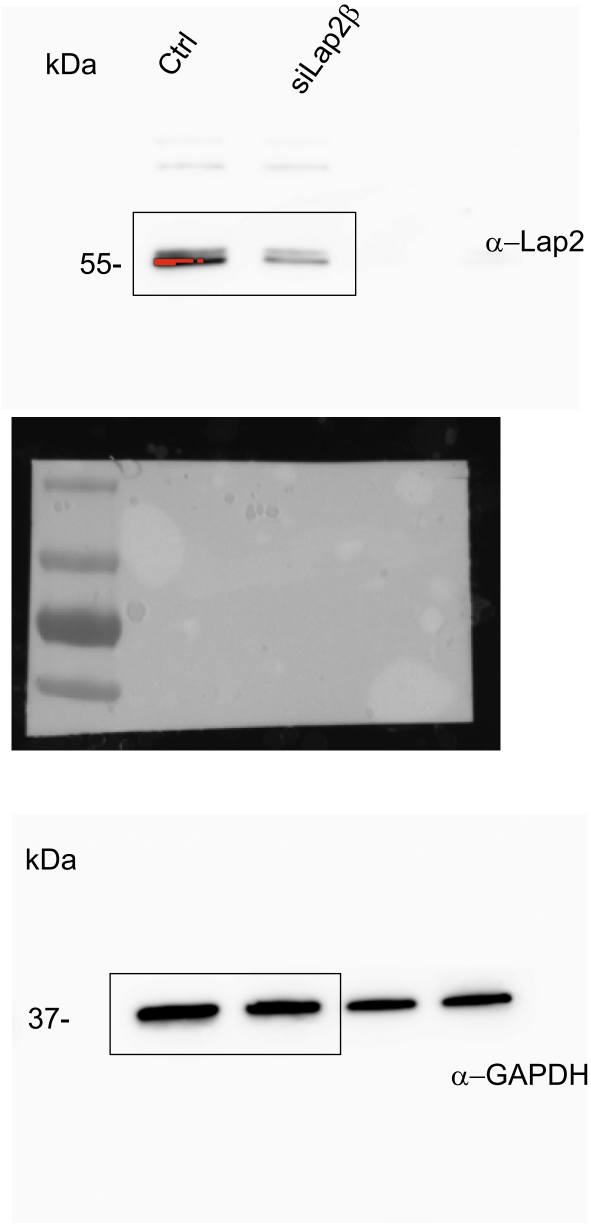

Supplementary 1i

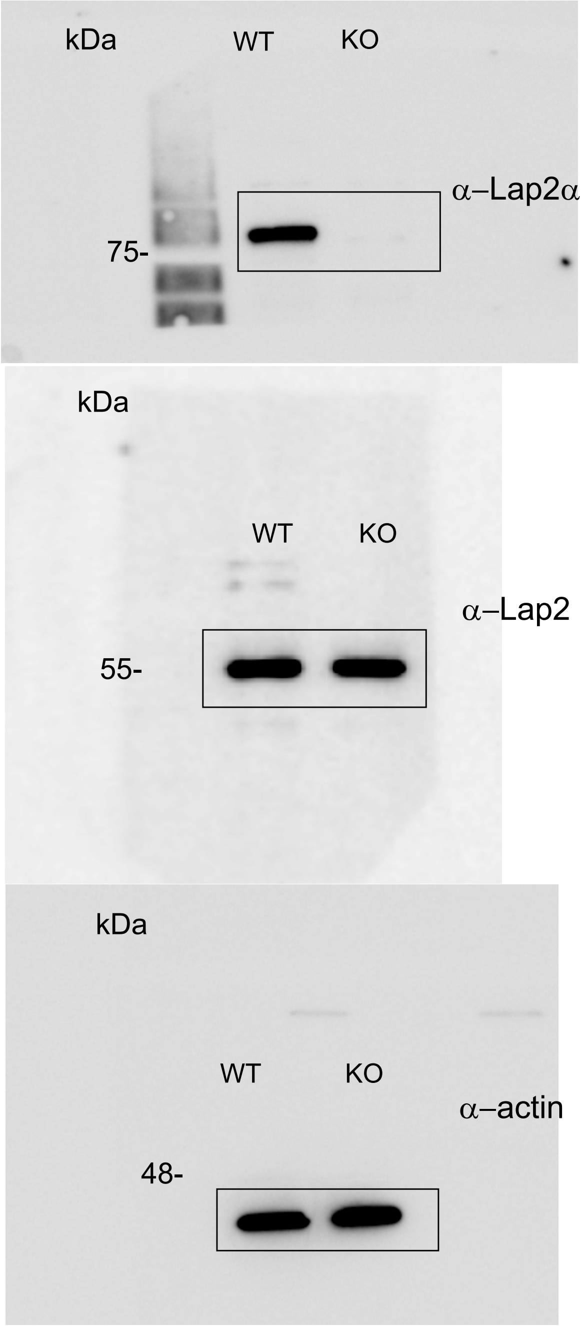

Supplementary 6a

inputs

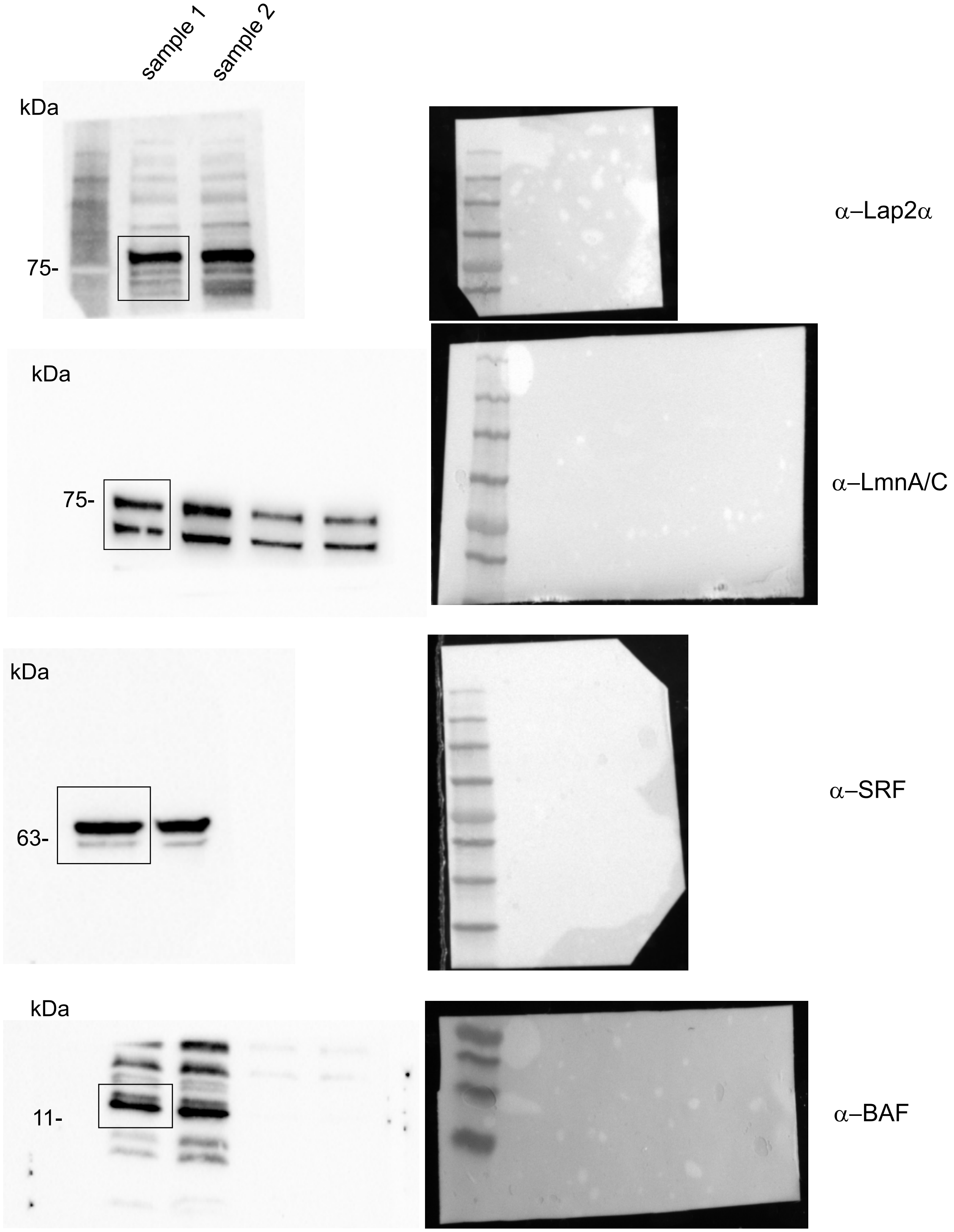

Supplementary 6a

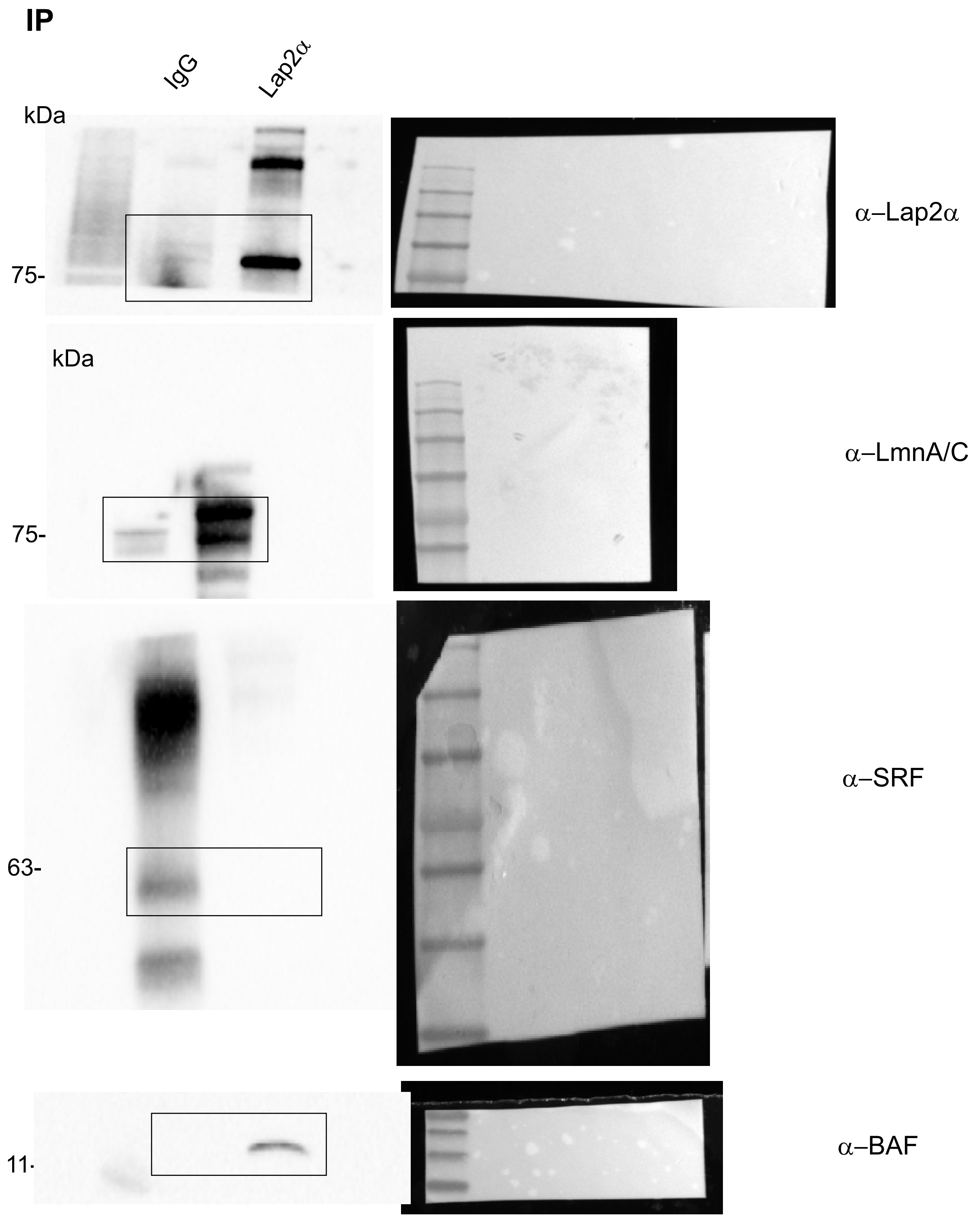

Supplement: Supplementary file 2 — Supplementary Figures. [file 41598_2022_6135_MOESM2_ESM.pdf]
